# Silibinin Downregulates the NF-κB Pathway and NLRP1/NLRP3 Inflammasomes in Monocytes from Pregnant Women with Preeclampsia

**DOI:** 10.3390/molecules24081548

**Published:** 2019-04-19

**Authors:** Mariana Leticia Matias, Virginia Juliani Gomes, Mariana Romao-Veiga, Vanessa Rocha Ribeiro, Priscila Rezeck Nunes, Graziela Gorete Romagnoli, Jose Carlos Peracoli, Maria Terezinha Serrao Peracoli

**Affiliations:** 1 Botucatu Medical School, Sao Paulo State University, UNESP, Botucatu 18618-691, Sao Paulo, Brazil; maromao14@gmail.com (M.R.-V.); va.rocharibeiro@gmail.com (V.R.R.); priscilarezeck@gmail.com (P.R.N.); jperacoli@uol.com.br (J.C.P.); 2 Institute of Biosciences of Botucatu, Sao Paulo State University, UNESP, Botucatu 18618-691, Sao Paulo, Brazil; vi.juliani@hotmail.com (V.J.G.); graziela.romagnoli@unesp.br (G.G.R.); terezinha.peracoli@unesp.br (M.T.S.P.)

**Keywords:** NLRP1/NLRP3 inflammasomes, NF-κB, monocytes, monosodium urate, preeclampsia, silibinin

## Abstract

Preeclampsia (PE) is a human pregnancy-specific syndrome with abnormal activation of cells from the innate immune system. The present study evaluated whether silibinin (SB) treatment of monocytes from preeclamptic women could modulate NLRP1 and NLRP3 inflammasomes as well as TLR4/NF-κB pathway activation. Peripheral blood monocytes from 20 preeclamptic and 20 normotensive (NT) pregnant women, as well as the THP-1 cell line, were cultured with or without monosodium urate (MSU) or SB. *NLRP1*, *NLRP3*, *Caspase-1*, *TLR4*, *MyD88*, *NF-κB*, *IL-1β*, *IL-18*, *TNF-α* and *IL-10* gene expression by monocytes was analysed by quantitative real-time polymerase chain reaction (qPCR), while inflammatory cytokine production and p65NF-κB activity were determined by enzyme-linked immunosorbent assays (ELISAs). TLR4/MyD88/NF-κB and NLRP1/NLRP3 inflammasomes pathways in THP-1 cells were evaluated by flow cytometry and western blot respectively. Compared with NT women, monocytes from preeclamptic women showed The Ethics Committee of the Botucatu Medical School approved the study (protocol number 2.333.216)higher endogenous activation of NLRP1/NLRP3 inflammasomes and the TLR4/NF-κB pathway as well as higher gene and protein expression of IL-1β, IL-18 and TNF-α, and lower expression of IL-10. Monocyte stimulation with MSU increased inflammation-related genes as well as NF-κB activity. In vitro, SB treatment of monocytes from preeclamptic women reduced the basal activation of these cells by decreasing NLRP1/NLRP3 inflammasomes and p65NF-κB activity. THP-1 cells exhibited a similar immunological response profile to monocytes from preeclamptic women when cultured with or without MSU or SB. These results suggest uric acid participates in the systemic inflammatory response characteristic of preeclampsia and that in vitro SB treatment can modulate the sterile inflammation established in monocytes from preeclamptic women.

## 1. Introduction

Silymarin is a flavonoid complex extracted from the seeds and fruit of *Silybum marianum*, a milk thistle plant that belongs to the family Asteraceae. It is one of the oldest medicinal herbs, and it is used as anti-inflammatory and hepatoprotective agent [1,2]. Silibinin (SB) is the main (70–80%) biologically active component of silymarin and also presents anti-inflammatory, hepatoprotective, anti-fibrotic, anticarcinogenic, antioxidant and immunomodulatory activities. Antioxidant and anti-inflammatory SB properties were demonstrated by dose-dependent inhibition of hydrogen peroxide (H_2_O_2_) release as well as production of tumour necrosis factor alpha (TNF-α), interleukin-10 (IL-10), transforming growth factor beta (TGF-β) and prostaglandin E2 (PGE2) by peripheral blood monocytes from healthy individuals stimulated with lipopolysaccharide (LPS) [3,4]. The SB anti-inflammatory effects are attributed to its suppression of nuclear factor kappa-B (NF-κB)-regulated gene products [3,5,6]. Thus, NF-κB plays a role in the pathogenesis of inflammation, a fact that suggests that NF-κB pathway inhibitors might be effective targets for treatment of chronic inflammatory diseases [6].

Preeclampsia (PE) is a specific human syndrome of pregnancy with multisystem involvement; it is considered the leading cause of maternal and foetal morbidity and mortality [7,8]. This pathology is identified primarily by onset of clinical parameters such as hypertension and proteinuria from 20 weeks of gestation or by hypertension associated with maternal neurologic or hematologic complications, kidney failure, liver involvement or foetal growth restriction [9,10,11]. Due to the characteristic systemic inflammation in PE, there is excessive activation of inflammatory cells such as monocytes and granulocytes and exacerbated production of pro-inflammatory cytokines [12,13] in addition to reduced production of regulatory cytokines such as IL-10 and TGF-β [14]. Monocytes from preeclamptic women are endogenously activated and therefore release significantly higher TNF-α, superoxide anion (O_2_^−^) and H_2_O_2_ compared to monocytes from normotensive (NT) pregnant women. These results suggest that PE is marked by oxidative stress and that the monocytes from maternal peripheral blood might be an important source of free radicals and inflammatory cytokines, all of which play a role in the pathogenesis of this disease [13,15].

Many preeclamptic women also show hyperuricemia associated with proteinuria and disease severity, an observation that suggests uric acid may contribute to PE pathogenesis through its inflammatory effects [15,16]. Uric acid induces inflammation and endothelial dysfunction [17], and its crystals (monosodium urate–MSU) can activate the NLRP3 inflammasome, a multimeric protein complex that is important for processing and releasing of IL-1β and IL-18 [18,19]. This activation requires two steps. The first, priming, is the interaction of a “danger signal”, such as pathogen-associated molecular patterns (PAMPs) or damage-associated molecular patterns (DAMPs), with toll-like receptors (TLRs), and subsequent NF-κB pathway activation, which results in increased transcription of NLRP3 and pro-IL-1β as well as apoptosis-associated speck-like protein (ASC) [20]. In the second, or triggering step, DAMP molecules interact with nod-like receptors (NLRs), which results in oligomerisation of NLRP3, ASC, and pro-caspase-1. When activated, pro-caspase-1 produces caspase-1, and its activity leads to the biologically active IL-1β and IL-18 that will be secreted into the extracellular medium [21,22].

In previous studies, we demonstrated that SB inhibits NF-κB pathway activation and pro-inflammatory cytokine production, including IL-1β and TNF-α, in peripheral blood mononuclear cells (PBMCs) from pregnant women with preeclampsia [14,23]. Moreover, monocytes from preeclamptic women showed endogenous activation of NLRP3 inflammasomes, and treatment of these cells with MSU enhances activation of this inflammatory complex [24]. SB treatment of pregnant rats in an experimental model of preeclampsia, induced by nitric oxide synthase inhibition with N-omega-nitro-l-arginine methyl (l-NAME), improves reproductive outcome, normalises blood pressure and platelet count and reduces proteinuria and serum levels of pro-inflammatory cytokines [25].

Considering the SB anti-inflammatory properties, the present study investigated whether SB exerts a modulatory effect on NLRP1 and NLRP3 inflammasomes and NF-κB expression in monocytes obtained from pregnant NT or preeclamptic women stimulated, in vitro, with MSU and treated with SB. We also evaluated whether THP-1 cells, a human monocyte cell line, have the same immunological response profile as these monocytes after treatment with MSU and SB.

## 2. Results

### 2.1. Clinical Characteristics

Analysis of the clinical characteristics of preeclamptic and NT pregnant women (Table 1) showed no statistical difference in maternal age or gestational age between the groups. Systolic and diastolic blood pressure, as well as proteinuria and uric acid concentration, were significantly higher in the preeclamptic compared to the NT group (*p* < 0.05).

### 2.2. Gene Expression in Monocytes Cultured with MSU or SB 

**Figure 1:**
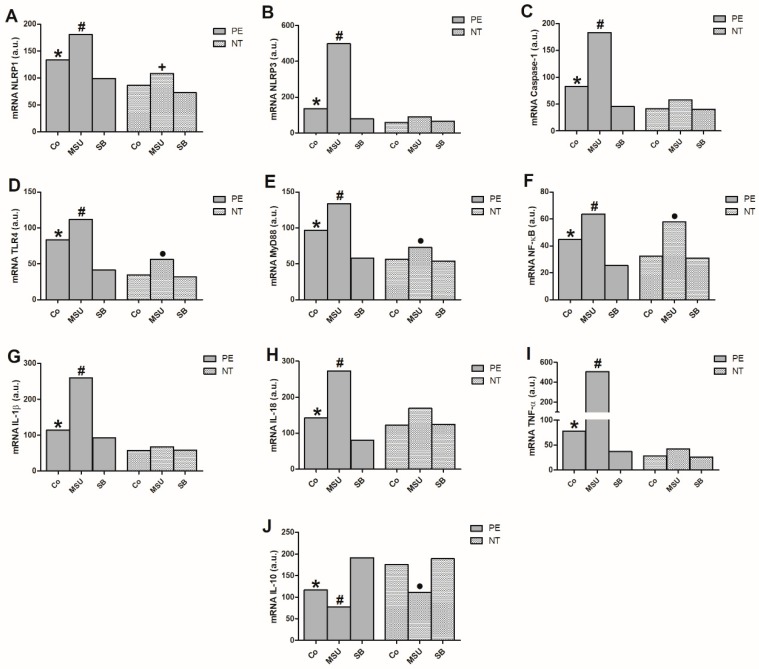
Gene expression of NLRP1 (**A**), NLRP3 (**B**), Caspase-1 (**C**), TLR4 (**D**), MyD88 (**E**), NF-κB (**F**), IL-1β (**G**), IL-18 (**H**), TNF-α (**I**) and IL-10 (**J**) in monocytes from 20 pregnant women with preeclampsia (PE) and 20 NT pregnant women, cultured in the absence (Co) or presence of MSU or SB. Results are expressed as the median in arbitrary units (a.u.). * *p* < 0.05 vs. NT Co, PE SB; # *p* < 0.05 vs. PE Co, PE SB; + *p* < 0.05 vs. NT SB; • *p* < 0.05 vs. NT Co, NT SB (Kruskal-Wallis test).

Basal *NLRP1*, *NLRP3*, *Caspase-1*, *TLR4*, *MyD88*, *NF-κB*, *IL-1β*, *IL-18* and *TNF-α* gene expression (Figure 1A–I) significantly increased in monocytes from women with PE compared to NT women.

Additionally, the endogenous gene expressions were higher than those exhibited by cultures of monocytes treated with SB in the preeclamptic group. Stimulating cells with MSU significantly enhanced activation of inflammasome-related genes (*NLRP1*, *NLRP3* and *Caspase-1*), *TLR4*, *MyD88* and NF-κB-pathway-related genes and inflammatory cytokine genes (*IL-1β*, *IL-18* and *TNF-α*) when compared to non-stimulated monocytes or those treated with SB in the preeclamptic group. There was no significant difference in *NLRP3* and *Caspase-1* expression or the inflammatory cytokines in monocytes from NT pregnant women stimulated with MSU or treated with SB. However, MSU induced higher *NLRP1*, *TLR4*, *MyD88* and *NF-κB* expression compared with control and SB-treated cultures for this group (Figure 1A,D–F).

Endogenous *IL-10* expression by monocytes from pregnant preeclamptic women was significantly lower compared to NT pregnant women monocytes as well as and to monocyte cultures treated with SB from the PE group (Figure 1J). After MSU stimulation, monocytes from preeclamptic women exhibited lower *IL-10* expression compared to non-stimulated and SB-treated cultures. Thus, SB treatment effectively increased *IL-10* expression in monocytes from preeclamptic women. In the NT group, basal monocyte *IL-10* expression was significantly higher compared to that obtained after MSU stimulation. IL-10 expression was not affected in NT monocytes treated with SB.

### 2.3. Determination of NF-κB in Monocyte Nuclear Extracts

**Figure 2:**
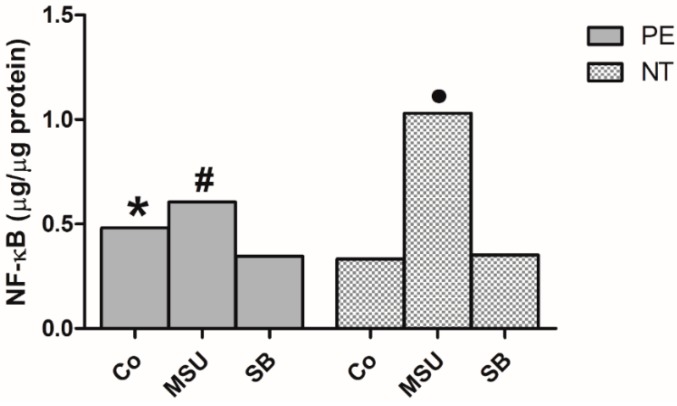
NF-κB concentration in nuclear extracts of monocytes from 20 pregnant women with preeclampsia (PE) and 20 NT pregnant women, cultured in the absence (Co) or presence of MSU or SB. Results are expressed as the median. * *p* < 0.05 vs. NT Co, PE SB; # *p* < 0.05 vs. PE Co, PE SB; • *p* < 0.05 vs. NT Co, NT SB (Kruskal-Wallis test).

Monocytes from pregnant women with PE showed significantly higher basal activation of the nuclear transcription factor NF-κB compared with those from NT pregnant women (Figure 2). After monocyte stimulation with MSU, both preeclamptic and NT women showed a significant increase in the amounts of NF-κB compared to non-stimulated (Co) and SB-treated monocytes. Treatment with SB decreased NF-κB levels in monocytes from preeclamptic group, but not in the NT group.

### 2.4. Cytokine Production by Monocytes from Pregnant Women

**Figure 3:**
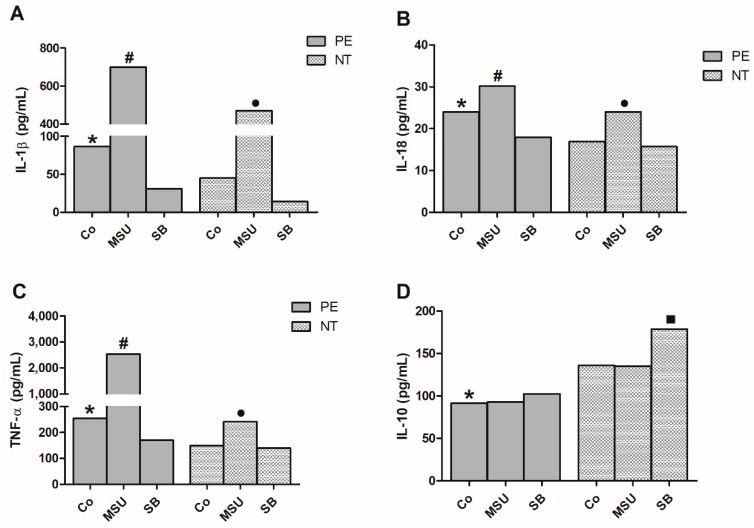
IL-1β (**A**), IL-18 (**B**), TNF-α (**C**) and IL-10 (**D**) production by monocytes. Monocytes from pregnant women with preeclampsia (PE) and NT pregnant women were cultured in the absence (Co) or presence of MSU or SB. Results are shown as the median. * *p* < 0.05 vs. NT Co, PE SB; # *p* < 0.05 vs. PE Co, PE SB; • *p* < 0.05 vs. NT Co, NT SB; ■ *p* < 0.05 vs. NT Co, NT MSU (Kruskal-Wallis test).

Figure 3 presents the production of TNF-α, IL-1β, IL-18 and IL-10 by monocytes from preeclamptic and NT pregnant women, cultured in the absence or presence of MSU or SB. There was a significant increase in the endogenous IL-1β, IL-18 and TNF-α concentrations produced by monocytes from pregnant preeclamptic compared to NT women. MSU stimulation induced higher IL-1β, IL-18 and TNF-α production by cells from both groups when compared to their respective control (Co), and to monocytes treated with SB. SB treatment of monocytes from the preeclamptic group significantly decreased IL-1β, IL-18 and TNF-α production when compared to the control culture. In NT group this effect was only observed in IL-1β production.

Basal IL-10 production in monocyte cultures from the PE group was significantly lower than in NT cultures. MSU treatment did not affect IL-10 production in both groups studied, whereas SB enhanced IL-10 in PE and NT cultures.

### 2.5. Analysis of the Expression of NF-κB Pathway in Monocytes

**Figure 4:**
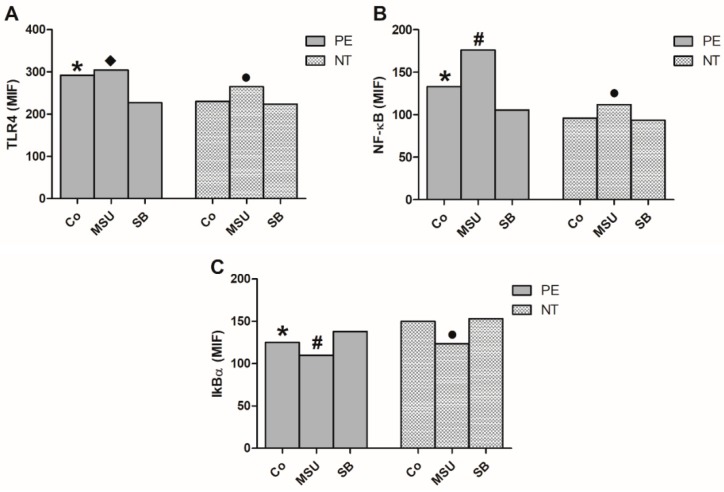
TLR4 (**A**), NF-κB (**B**) and IκBα (**C**) expression by monocytes. Monocytes from pregnant women with preeclampsia (PE) and NT pregnant women were cultured in the absence (Co) or presence of MSU or SB. Results are shown as the median. *p < 0.05 vs. NT Co, PE SB; # *p* < 0.05 vs. PE Co, PE SB; • *p* < 0.05 vs. NT Co, NT SB; ♦ *p* < 0.05 vs. PE SB (Kruskal-Wallis test).

Flow cytometric analysis showed that monocytes from pregnant women with PE presented higher endogenous expression of TLR4 and NF-κB and lower expression of IκBα when compared to NT pregnant women (Figure 4). Monocyte culture of preeclamptic women with MSU did not increase TLR4 expression by these cells. However, treatment with SB decreased its level (Figure 4A). Regarding the group of NT pregnant women, monocytes cultured in the presence of MSU showed higher TLR4 and NF-κB expression in relation to control (Co) and SB-treated cultures (Figure 4A,B). The protein level expression of NF-κB increased due to the addition of MSU in the culture of monocytes from pregnant women with PE, whereas treatment with SB decreased these levels (Figure 4B). Monocytes from preeclamptic and NT pregmant women, cultured in the presence of MSU, had decreased IκBα expression, while SB treatment caused an increase in this factor. (Figure 4C).

### 2.6. Gene Expression in THP-1 Cells Cultured with MSU or SB 

Relative quantification of *NLRP1*, *NLRP3*, *Caspase-1*, *TLR4*, *MyD88*, *NF-κB*, *IL-1β*, *IL-18*, *TNF-α* and *IL-10* expression was performed in THP-1 cells that were either stimulated with MSU or stimulated with MSU and treated with SB.

**Figure 5:**
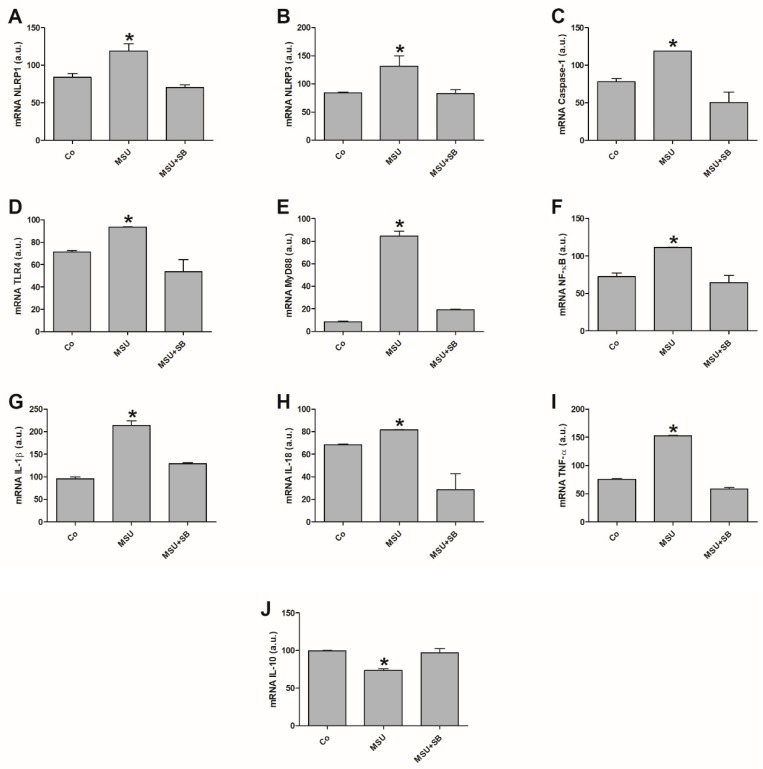
Gene expression of NLRP1 (**A**), NLRP3 (**B**), Caspase-1 (**C**), TLR4 (**D**), MyD88 (**E**), NF-κB (**F**), IL-1β (**G**), IL-18 (**H**), TNF-α (**I**) and IL-10 (**J**) in THP-1 cells cultured in the absence (Co) or presence of MSU or SB. Results, obtained from five independent experiments, are shown as mean ± standard deviation (SD) in arbitrary units (a.u.). * *p* < 0.05 vs. Co and MSU + SB (ANOVA).

MSU-stimulated THP-1 cells had higher expression of inflammasome genes (*NLRP1*, *NLRP3*, *Caspase-1*, *IL-1β* and *IL-18*) when compared to the control (Co) and MSU+SB-treated cultures (Figure 5A–C,G,H). Similar results were observed for *TLR4*, *MyD88* and *NF-κB* expression in these cells (Figure 5D–F). *TNF-α* was significantly increased in MSU-stimulated THP-1 cells compared to the control and MSU+SB cultures (Figure 5I). Additionally, the anti-inflammatory cytokine *IL-10* expression was significantly lower in THP-1 cells stimulated with MSU compared to the control culture and cultures treated with SB (Figure 5J).

### 2.7. Cytokine Production by THP-1 cells Cultured with MSU and/or SB

**Figure 6:**
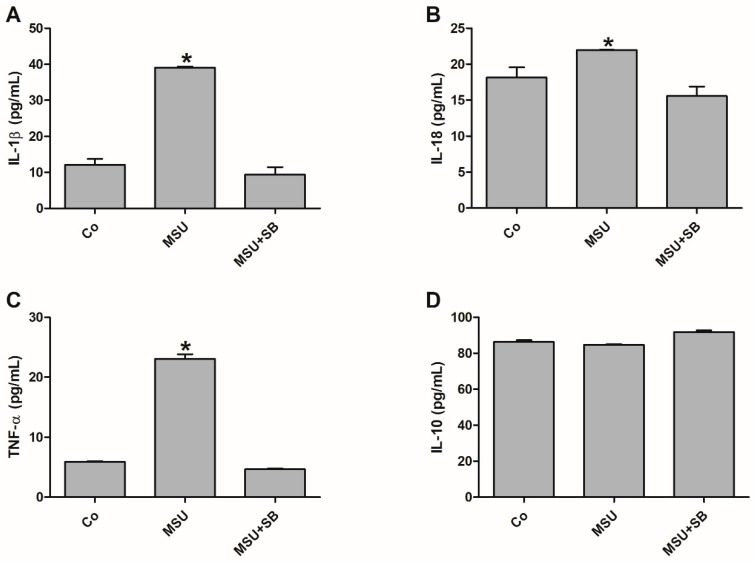
IL-1β (**A**), IL-18 (**B**), TNF-α (**C**) and IL-10 (**D**) production by THP-1 cells cultured in the absence (Co) or presence of MSU or silibinin (SB). Results, obtained from five independent experiments, are shown as mean ± SD in arbitrary units (a.u.). * *p* < 0.05 vs. Co, MSU + SB (ANOVA).

THP-1 cells stimulated with MSU produced increased concentrations of IL-1β, IL-18 and TNF-α when compared to the control and MSU + SB-treated cultures. There were no significant diferences between THP-1 cultures with regards to IL-10 production (Figure 6).

### 2.8. Inflammasomes Protein Componentes in THP-1 Cells Cultured with MSU and/or SB

**Figure 7:**
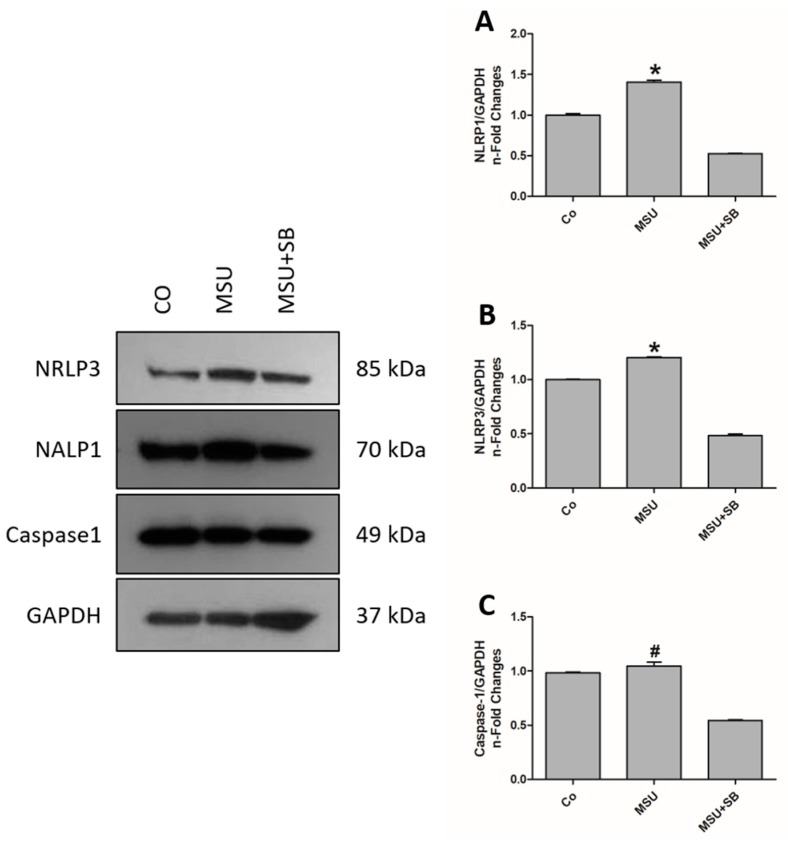
NLRP1 (**A**), NLRP3 (**B**) and Caspase-1 (**C**) protein expression by THP-1 cells cultured in the absence (Co) or presence of MSU or silibinin (SB). Results, obtained from five independent experiments, are shown as mean ± SD in arbitrary units (a.u.). * *p* < 0.05 vs. Co, MSU + SB; # *p* < 0.05 vs. MSU + SB (ANOVA).

THP-1 cells cultured with MSU produced increased concentrations of NLRP1 and NLRP3 when compared to the control and MSU + SB-treated cultures. Caspase-1 production did not increased after MSU stimulation, but it decreased significantly in cultures treated with MSU + SB (Figure 7).

## 3. Discussion

The results of the present study show that monocytes from preeclamptic women are endogenously activated and may play a role in the systemic inflammatory response already described in PE [26,27]. Compared with NT pregnant women, basal gene expression of NLRP1 and NLRP3 inflammasomes, the TLR4/NF-κB pathway and pro-inflammatory cytokines were significantly increased in monocytes from preeclamptic women, findings that highlight the inflammatory profile of these cells.

Activation of monocytes from preeclamptic patients includes high expression of surface markers such as TLR4 and CD64, increased production of pro-inflammatory cytokines and elevated oxygen free radical release compared with NT pregnant women [15,28,29]. The activation state of these cells seems to be related to DAMPs, circulating molecules capable of inducing sterile inflammation [30]. Several DAMPs, such as uric acid [24], heat shock proteins (e.g., Hsp70) [31], products released from the extracellular matrix (e.g., hyaluronan) [32] and proteins released from damaged or stressed cells (e.g., high mobility group box-1 (HMGB1)) [33], are elevated in preeclamptic patient plasma and may interact with TLRs or nod-like receptors with a pyrin domain (NLRP), actions that lead to monocyte activation in PE [27].

The results of significantly higher uric acid plasma concentrations in preeclamptic compared to NT pregnant women, detected in the present study, confirms previous reports [15,24]. The origin of this DAMP in plasma is unknown, but it is possible that hyperuricemia may induce inflammasomes and activate NF-κB, changes that would result in an exaggerated inflammatory state in PE. Previous studies reported uric acid effects on the innate immune system, such as its potential to activate the inflammasome complex [18]. Our data corroborate previous results showing that monocyte stimulation with MSU increases gene expression of inflammasome components in preeclamptic women [24]. In addition, in the present study, the activation of TLR4/MyD88/NF-κB and NLRP1/NLRP3 inflammasomes pathways were also detected after MSU-monocyte stimulation, results so far not yet demonstrated in PE. NF-κB pathway activation is essential to upregulate pro-IL-1β and NLRP3 protein synthesis [34]. Furthermore, MSU stimulus increased *IL-1β* and *TNF-α* mRNA levels, results that corroborate the literature [24]. On the other hand, the elevated endogenous production of TNF-α by monocytes from preeclamptic women might act in inflammasome complex activation by stimulating the NF-κB pathway and inducing *TNF-α* and *IL-1β* transcription [35]. In turn, these cytokines may act by promoting NF-κB activation, which would consequently maintain a cycle of cellular activation [36] that results in the excessive and chronic inflammation observed in PE.

In the present study, diminished basal inflammatory gene expression for *NLRP1*, *NLRP3*, *Caspase-1*, *TLR4*, *MyD88*, *NF-κB*, *IL-1β*, *IL-18* and *TNF-α* by monocytes from NT pregnant women could be associated with IL-10 regulatory activity, namely once its gene and protein expression are significantly higher than in monocytes from the PE group. Low IL-10 production by monocytes occurs in patients with PE [14,29]. Thus, high IL-10 production plays beneficial roles in normal pregnancy by promoting successful placentation and controlling excessive inflammation through downregulation of *IL-1β* and *TNF-α* gene expression [37,38].

Our results demonstrated that in vitro treatment of monocytes from preeclamptic women with SB induced *IL-10* expression, a finding which confirms the anti-inflammatory effect of this flavonoid [3,6,23]. Moreover, in monocytes from the PE group, SB treatment reduced the endogenous activation of inflammasome (*NLRP1*, *NLRP3* and *caspase-1*) and NF-κB-pathway-related (*TLR4*, *MyD88* and *NF-κB*) gene expression and reduced NF-κB activation and its nuclear extract concentration. Regarding cytokine production, SB treatment reduced *IL-1β*, *IL-18* and *TNF-α* mRNA and protein levels in monocytes from preeclamptic women. These polyphenolic flavonoid effects might be related to a SB suppressive effect on the phosphorylation of IκB, a protein bound to NF-κB in the cytoplasm that prevents its migration to the nucleus and impairs the transcription of inflammation-related genes [39,40]. Thus, SB can modulate inflammatory effects in these cells even when administrated in an already inflammatory environment.

Activation of the NLRP1 and NLRP3 inflammasomes, the NF-κB pathway and inflammatory cytokines by MSU, as well as their modulation by SB, were reproduced in the THP-1 human monocyte cell line. We demonstrated that MSU activated THP-1 cells and, consequently, triggered inflammasome and NF-κB pathway activation. On the other hand, THP-1 cells stimulated with MSU and treated with SB showed a marked reduction in their inflammatory profile, a change seen by diminished inflammation-related gene expression of NLRP1/NLRP3 inflammasomes and TLR4/NF-kB pathway as well as inflammatory cytokine production. These results show that SB plays a role as a potent anti-inflammatory agent, by regulating NF-κB signaling and NLRP3 inflammasome activation. The inhibitory effect of SB on NF-κB activation is well known (3,5,6), but studies evaluating its effects on NLRP3 inflammasome activation are scarce. Recent experimental studies showed that SB exerts protective effect against lung injury and nonalcoholic fat liver disease by inhibitory effect on NF-κB and NLRP3 inflammasome pathways. In vitro treatment of THP-1 cells with SB led to inhibition of caspase-1 cleavage and IL-1β and TNF-α production [41]. SB also inhibits NLRP3 inflammasome assembly through NAD+/SIRT2 pathway in mice with non-alcoholic fat liver disease [42].

In conclusion, the high serum levels of uric acid detected in preeclamptic women, suggests that these crystals, acting as a circulating DAMP, participates in the excessive inflammation present in PE by inducing activation of NLRP1/NLRP3 inflammasomes and the NF-κB pathways in monocytes from these patients. SB treatment modulated the sterile inflammation established in monocytes from pregnant women with PE, an effect that demonstrate its relevant role on the regulation of the inflammatory response in this disease. The employment of THP-1 cells in this study confirms the anti-inflammatory activity of SB on cells stimulated with MSU. Considering these results, as well as the existing clinical uses and notorious safety of this flavonoid, future studies on SB immunomodulatory effects on the exacerbated inflammation characteristics of PE are encouraged to further elucidate its potential as an adjuvant treatment for this gestational pathology.

## 4. Materials and Methods

### 4.1. Subjects

The study comprised 40 pregnant women without a previous history of hypertension or obstetric and medical complications, admitted to the Obstetric Unit of Botucatu Medical School, Botucatu (SP, Brazil). Twenty women were diagnosed with PE, defined as a persistently elevated blood pressure of 140 over 90 mmHg and proteinuria (≥300 mg in urine collected during 24 h) after the twentieth week of gestation, or in the absence of proteinuria, by new-onset hypertension associated with other complications such as thrombocytopenia, HELLP (hemolysis, elevated liver enzymes, low platelet count) syndrome, new-onset cerebral or visual disturbances, renal insufficiency or acute pulmonary edema [9]. A group of 20 pregnant women with an uncomplicated pregnancy who remained NT and non-proteinuric were recruited as controls and matched for gestational age with the preeclamptic group. Gestational age was calculated from the last menstrual period and confirmed by early (<12 weeks gestation) ultrasound examination. Proteinuria in 24-h urine was measured by a colorimetric method, the Technicon RA-XT automation system (Miles Inc., Tarrytown, NY, USA) and uric acid was assessed by uric acid enzymatic Trinder (Biotrol Diagnostic, Chennevières-lès-Louvres, France) in the Clinical Laboratory of Botucatu Medical School–UNESP. Exclusion criteria included prior preeclampsia, multiple gestation, illicit drug use and pre-existing medical conditions such as diabetes, chronic hypertension and infectious and renal disease. The Ethics Committee of the Botucatu Medical School approved the study (protocol number 2.333.216), and all women gave written informed consent. Parents or guardians signed for women aged less than 18 years.

### 4.2. Blood Sampling

For the evaluation of gene expression, cytokine production and NF-κB quantification in monocytes from women with PE, blood was collected at the time of disease diagnosis, and from NT pregnant women at the time when they were matched for gestational age with women with PE. Blood samples (10 mL) were collected by venipuncture from the antecubital vein and were put into a sterile plastic tube that contained 10 U/mL ethylenediaminetetraacetic acid (EDTA; Becton Dickinson-BD Vacutainer; BD Biosciences, Franklin Lakes, NJ, USA). After blood centrifugation at 4 °C for 10 min at 1200 g, the plasma fraction was removed and aliquots were stored at −80° until uric acid determination.

### 4.3. Monocyte Cultures

After plasma separation, PBMCs were isolated by density gradient centrifugation on Ficoll-Paque Premium [density (d) = 1.077] (GE Healthcare Bio-Sciences, Uppsala, Sweden) as previously described [15]. Cell viability, as determined by 0.2% trypan blue (Gibco) dye exclusion, was >95% in all experiments. Monocytes were counted using neutral red (0.02%) in the PBMC suspension, and 5 × 10^5^ monocytes/mL in complete medium were distributed (1 mL/well) in 24-well flat-bottomed plates (NalgeNunc, Rochester, NY, USA). After incubation for 2 h at 37 °C in a humidified 5% CO_2_ atmosphere, non-adherent cells were removed by aspiration and each well rinsed twice with complete medium. Monocyte preparations routinely contained >90% monocytes as determined by morphologic examination and staining for non-specific esterase [43]. Monocytes were incubated with complete medium in the presence or absence of 50 µg/mL of MSU (Sigma-Aldrich, St. Louis, MO, USA) or 50 µM of SB (Sigma-Aldrich) for 18 h at 37 °C in 5% CO_2_. The MSU and SB concentrations used in monocyte cultures were previously standardized [23,24]. Culture supernatants were collected and stored at −80 °C until cytokines determination.

### 4.4. THP-1 Cell culture

THP-1 cells (ATCC No. TIB 202) were obtained from the collection of the Recombinant Technology Laboratory (LATER; Bio-Manguinhos, Fiocruz, Rio de Janeiro, RJ, Brazil) and maintained in liquid nitrogen (−196 °C) for a maximum of 12 months. Cells were thawed from cryotubes. Subsequently, they were washed three times with 1x phosphate buffered saline (PBS; 137 mM NaCl (Merck KGaA, Darmstadt, Germany), 2.7 mM KCl (Sigma-Aldrich), 10 mM Na_2_HPO_4_ (Merck) and 1.8 mM KH_2_PO_4_ (Merck)) pH 7.4. For maintenance, cells were cultured in RPMI 1640 (Sigma-Aldrich), formulated with 0.2% (*v*/*v*) NaHCO_3_ (Sigma-Aldrich) and supplemented with 20% (*v*/*v*) inactivated fetal bovine serum (Gibco), and grown in 25 cm^2^ T-bottles (Corning Incorporated, Corning, NY, USA) at 37 °C in 5% CO_2_ for 3 days. Cell concentration and viability were determined on a haemocytometer by light microscopy (100×) using a 1:50 dilution in 0.4% trypan blue.

For experimental preparations, cells were removed from the original culture medium, washed twice in PBS and resuspended in 1 ml of RPMI 1640 medium supplemented with 10% (*v*/*v*) fetal bovine serum. After quantification, the cells were suspended at a concentration of 5 × 10^5^ cells/ml in RPMI 1640 supplemented with 10% (*v*/*v*) fetal bovine serum, and were incubated with complete medium in the presence or absence of 50 µg/mL of MSU (Sigma-Aldrich) or 50 µg/mL of MSU + 50 µM of SB (Sigma-Aldrich) for 18 h at 37 °C in 5% CO_2_. Culture supernatants were collected and stored at −80 °C until cytokines determination.

### 4.5. Nuclear Extraction of Monocytes

Monocytes from preeclamptic and NT pregnant women, at a concentration of 5 × 10^5^ cells/mL, were cultured for 30 min in the presence or absence of 50 µg/mL MSU or 50 µM SB. The cells, obtained as described in the monocyte cultures section, were subjected to nuclear extraction using a nuclear extraction kit (Cayman Chemical Company, Ann Arbor, MI, USA) according to the manufacturer’s instructions.

### 4.6. Determination of p65NF-κB Activity

Nuclear extracts for each culture condition were employed to determine the p65NF-κB level by using a transcription factor enzyme-linked immunosorbent assay (ELISA) kit (Cayman Chemical) according to the manufacturer’s instructions. Total protein concentration in nuclear extracts was determined by Lowry’s method [44], and p65NF-κB was expressed as μg/μg of nuclear protein.

### 4.7. Cytokine Determinations

Cytokine concentrations in monocytes and THP-1 culture supernatants were determined by Quantikine ELISA kits (R&D Systems, Minneapolis, MN, USA) for TNF-α, IL-1β, IL-18 and IL-10 according to the manufacturer’s instructions. Assay sensitivity limits were 1.6 pg/mL for TNF-α, 1.0 pg/mL for IL-1β, 3.9 pg/mL for IL-10 and 2.25 pg/mL for IL-p18.

### 4.8. Expression of Transcripts Related to Inflammation

Monocytes and THP-1 cells were incubated with complete medium in the presence or absence of 50 µg/mL MSU or 50 µM SB for 4 h at 37 °C in 5% CO_2_. The cells were subjected to analysis of the transcriptional level of the genes that encode the proteins NLRP1, NLRP3, Caspase-1, TLR4, MyD88, NF-κB, IL-1β, IL-18, TNF-α and IL-10. Total RNA was extracted from monocytes with the Total RNA Purification Kit (NorgenBiotek Corp., Thorold, ON, Canada) according to the manufacturer’s protocol. After extraction, 1 µg of total RNA was incubated with DNase I Amp Grade (Invitrogen, ThermoFisher Scientific, Waltham, MA, USA). The purity and relative quality of samples were determined by fluorometry using Qubit^®^ Fluorometric Quantitation (ThermoFisher Scientific, Waltham, MA, USA). Subsequently, complementary DNA (cDNA) was synthesised in a 60 μL reaction (with 450 ng total RNA) using the ImProm-II^TM^ Reverse Transcription System, according to the manufacturer’s protocol. Quantitative real-time polymerase chain reaction (qPCR) was performed using RT GoTaq^®^ qPCR Master Mix (Promega, Madison, WI, USA) according to [24]. A 7500 Fast Real-Time PCR System (Applied Biosystems–ThermoFisher Scientific, Waltham, MA, USA) was used for analysis. Variants of the studied targets were aligned in the MEGA 5.1 program and each primer was subsequently selected by the software Primer-BLAST. Primers located in exon-exon junctions guarantee the purity of the reaction, namely the absence of any genomic DNA that may contaminate it. The primer sequences used in this study are shown in Table 2.

### 4.9. Expression of Intracytoplasmic Factors and TLR4 Receptor in Monocytes

Monocytes from pregnant women with PE and normotensive pregnant women were cultured in the presence or absence of 50 µg/mL MSU or 50 µM of SB for 30 minutes for the analysis of the expression of intracytoplasmic factors NF-κB and IκBα and for 18 h for the TLR4 surface receptor analysis. The cell concentration was adjusted to 2 × 10^5^ cells/mL and the cells were distributed in Falcon tubes for cytometer (BD Bioscience). Cells were incubated with BD Biosciences antibodies, with respective fluorochromes: anti-CD14 (Alexa FLuor 488) and anti-TLR4 (PE) for 30 min in the dark. After centrifugation, cells were washed twice with PBS containing 1% sodium azide and 1% fetal bovine serum and centrifuged again for 10 min at 400 g. The cells were then fixed and permeabilized using Fix Buffer (BD) and Perm Buffer (BD) for incubation with specific antibodies (BD Biosciences) labelled with specific fluorochromes for the intracellular proteins NF-κB (PE-Cy7) and IκBα (Alexa FLuor 647), for 30 min, in the dark and at room temperature. For each test, control tubes were incubated with specific isotypic antibodies for each fluorochrome (Alexa FLuor 488, PE, PE-Cy7 and Alexa Fluor 647). The analyzes were performed on a FACSCanto^TM^ (BD) flow cytometer using FlowJo program (FlowJo Enterprises, Ashland, OR, USA) to acquire and analyze cellular multiparameters. It was standardized the acquisition of 30,000 events per sample, and the population of interest was optimized, establishing a gate based on size (FSC) and granularity (SSC) or fluorescence (FL) parameters of CD14. From this gate, established on CD14 positive monocytes, the mean fluorescence intensity (MIF) of TLR4, NF-κB and IκBα was evaluated.

### 4.10. Evaluation of Proteins Related to Inflammasomes in THP-1 Cells

THP-1 cells, cultured for 18 h in the presence or absence of 50 µg/mL MSU or 50 µM SB, were subjected to cell lysis using lysis buffer consisting of 1 mM EDTA, 0.5% Triton X-100, 10 μg/mL leupeptin, 10 μg/mL pepstatin, 100 µM phenylmethylsulfonyl fluoride (PMSF) and 3 μg/mL Aprotinin in phosphate buffered saline (PBS) pH 7.2–7.4. The cell extract was centrifuged at 1500 g for 10 min and subjected to the determination of total proteins by the Lowry’s method [44].

After determining the protein concentration of the THP-1 cell lysate, 25 μg of protein were treated with gel run solution buffer (Bio-Rad, Hercules, CA, USA) and β-mercaptoethanol at 95 °C for 5 min. Then, the proteins were separated on SDS-PAGE and after electrophoresis, transferred to the nitrocellulose membrane. Nonspecific protein binding was blocked by incubating the membranes in 5% skim milk in Tris-HCl buffer containing 0.1% Tween 20 (TBST) for 45 min at room temperature. The membranes were subsequently incubated for 18 h with rabbit polyclonal antigen-specific anti-NLRP1, anti-NLRP3 and anti-caspase-1 antibodies (Novus Biologicals, Littleton, CO, USA) containing TBST, at the concentration recommended by the manufacturer. After washing, the membranes were incubated with rabbit-specific secondary antibody diluted in TBST for 1 h. The immunoreactive components were revealed by the Luminescent Kit (Amersham™ ECL Select™ Western Blotting Detection Reagent) purchased from GE Healthcare^®^.

### 4.11. Statistical Analysis

The clinical characteristics of pregnant NT or preeclamptic women, as well as the data on monocyte gene and proteic expression and NF κB quantification, were analysed by non-parametric methods (Kruskal-Wallis and Mann-Whitney U test). The data for THP-1 cellular gene and proteic expression were evaluated by parametric analysis of variance (ANOVA). Results were evaluated using the statistical program GraphPad Prism, version 6.01 (GraphPad Inc., San Diego, CA, USA), and statistical significance was accepted at *p* < 0.05.

## Figures and Tables

**Table 1:** Clinical and laboratory characteristics of preeclamptic and NT pregnant women.

Characteristics	Pregnant Women with Preeclampsia (*n* = 20)	NT Pregnant Women (*n* = 20)
Age (years)	26 (17–41)	27 (18–40)
Gestational age (weeks)	34 (23–39)	35 (23–40)
Systolic Blood Pressure (mmHg)	160 * (140–200)	110 (90–112)
Diastolic Blood Pressure (mmHg)	110 * (90–120)	69 (63–70)
Proteinuria (mg/24 h)	7250 * (300–18800)	<300
Uric acid (mg/dL)	6.2 * (3.9–10.1)	3.2 (2.3–4.7)

Data are presented as the median, with the minimum and maximum values in parentheses. * *p* < 0.05 vs. NT pregnant women (Mann-Whitney *U* test).

**Table 2:** Primers for inflammasome and NF-κB pathway components, cytokines and *GAPDH*. Numbers indicate nucleotide positions in the corresponding transcripts.

Gene	Forward Primer (5′–3′)	Reverse Primer (5′–3′)	GenBank
***NLRP1***	(1728)TCCGGCTCCCATTAGACAGA(1747)	(1810)AGACCCATCCTGGCTCATCT(1791)	NM_033004.3
***NLRP3***	(2826)GAGGAAAAGGAAGGCCGACA(2845)	(2917)TGGCTGTTCACCAATCCATGA(2897)	NM_004895.4
***CASP1***	(1065)AGACATCCCACAATGGGCTC(1084)	(1172)TGAAAATCGAACCTTGCGGAAA(1151)	NM_033292.3
***TLR4***	(2274)TGCTTCTTGCTGGCTGCATA(2293)	(2359)CCAGTCCTCATCCTGGCTTG(2340)	NM_138554.4
***MYD88***	(263)GTCTCCTCCACATCCTCCCT(282)	(344)TCCGCACGTTCAAGAACAGA(325)	NM_001172567.1
***NFKB1***	(1072) TGCAGCAGACCAAGGAGATG(1091)	(1211) TGCATTGGGGGCTTTACTGT (1192)	NM_003998.3
***IL1B***	(544)GAGCAACAAGTGGTGTTCTCC(564)	(653)AACACGCAGGACAGGTACAG(634)	NM_000576.2
***IL18***	(438)ACTGTAGAGATAATGCACCCCG(459)	(517)AGTTACAGCCATACCTCTAGGC(496)	NM_001562.3
***TNF***	(325)GCTGCACTTTGGAGTGATCG(344)	(462)GGGTTTGCTACAACATGGGC(443)	NM_000594.3
***IL10***	(361)AAGACCCAGACATCAAGGCG(380)	(445)ATTCGATGACAGCGCCGTAG(426)	NM_000572.2
***GAPDH***	(684)CGTGGAAGGACTCATGACCA(703)	(801)GGCAGGGATGATGTTCTGGA(782)	NM_002046.4
